# Child- and family impacts of infants’ oral conditions in Tanzania and Uganda– a cross sectional study

**DOI:** 10.1186/1756-0500-5-538

**Published:** 2012-09-28

**Authors:** Ray Masumo, Asgeir Bardsen, Kijakazi Mashoto, Anne Nordrehaug Åstrøm

**Affiliations:** 1 Department of Clinical Dentistry, Community Dentistry, University of Bergen, Bergen, Norway; 2 Centre for International Health, University of Bergen, Bergen, Norway; 3 Muhimbili University of Health and Allied Sciences, Dar Es Salaam, Tanzania

## Abstract

**Background:**

Early childhood dental caries impacts on the quality of life of children and their families. This study set out to assess the psychometric properties of an oral health related quality of life, OHRQoL, measure, based on items emanating from the Child-and Family impact sections of the Early Childhood Oral Health Impact Scale (ECOHIS), in Kiswahili and Luganda speaking communities. It was hypothesized that the Child- and Family impact scores would discriminate between children with and without clinically defined dental problems and reported good and bad oral health.

**Method:**

Kiswahili and Luganda versions of the Child- and Family impact scores were derived through translation in pilot studies. Totals of 1221 and 816 child/caretaker pairs attending health care facilities in Manyara, Tanzania and Kampala, Uganda, were recruited into the study. After caretakers completed the interview, their children underwent oral clinical examination.

**Results:**

Internal consistency reliability (Cronbach’s alpha) was > 0.80 with respect to the Child impact score and 0.79 regarding the Family impact score. Multiple variable logistic- and Poisson regression analyses revealed that the Kiswahili and Luganda versions of the Child- and Family impact score associated in the expected direction with child’s oral diseases as with their reported health and oral health status. In Manyara, multiple logistic regression revealed that the ORs of reporting Child impacts were 1.8 (95% CI 1.0-3.4) and 2.2 (1.3-3.4) among caretakers who confirmed linear hypoplasia and teething symptoms, respectively. In Kampala, the ORs for reporting Child impacts were 2.3 (95% CI 1.3-3.9), 1.7 (95% CI 1.1-2.5), 1.6 (95% CI 1.2-2.3) and 2.7 (95% CI 1.3-5.8) among those who confirmed teeth present, hypoplasia, teething symptoms and tooth bud extractions, respectively. The odds ratios for reporting Family impacts were 2.7 (95% CI 1.5-4.7), 1.5 (95% CI 1.1- 2.1) and 4.6 (95% CI 2.0-10.7) if reporting LEH, teething symptoms and toothbud experience, respectively.

**Conclusion:**

The Child and Family impact scores demonstrated acceptable internal consistency reliability and reproducibility whereas the discriminative validity was more ambiguous. The OHRQoL scores should be developed further and tested among Kiswahili and Luganda speaking caretakers.

## Background

Growing global concerns about the consequences of children’s functional status coincides with the development and testing of disability- and health related quality of life measures [1,2]. Socio-dental indicators to assess the quality of life impacts of children’s oral conditions have been developed for school-agedÂ children considering their cognitive, social, and emotional developmental stages [3-6]. Most measures contain items pertaining to children themselves and their parents [6]. Yet, there are relatively few oral health related quality of life (OHRQoL) measures designed for pre-schoolchildren, although evidence suggests that 0–5 year olds encounter oral diseases, such as early childhood caries (ECC), teething symptoms, dental trauma and linear enamel hypoplasia, (LEH), that impact negatively on their functional, psychological and social well- being [7,8]. Left untreated, ECC impacts on quality of life to an extent similar to other systemic diseases and might lead to dental pain, avoidance of certain types of foods and might interfere adversely with anthropometric and nutritional status, socializing, self-esteem and learning abilities [9]. ECC might also lead to widespread interruptions of family functioning and restrict daily activities for parents/caregivers, such as lost workdays for caregivers who have to stay home with their child or spend time and money to access dental care [10]. In line with a rising global focus on child’s disability and participation in clinical research, the necessity of valid OHRQoL measures for children is growing.

The concept of OHRQoL relates to the impact of oral diseases on individuals’ daily functioning, well- being and quality of life [2]. Theoretically, OHRQoL is a function of various symptoms and experiences representing individuals’ subjective perceptions. Presently, the ECOHIS is one of only a few measures developed for use in preschool children [8,11-15]. In line with other OHRQoL measures, ECOHIS is based on an explicit conceptual framework; the World Health Organizations’ International Classification of Impairments, Disabilities and Handicaps [16]. According to recent developments, OHRQoL is recognized to be an interaction between individual health condition and contextual factors, recognizing the influence of environments and access to dental care [2]. ECOHIS was originally developed in English, and has been adapted for use in other languages, including French, Chinese, Brazilian and Farsi [11-15]. This inventory is administered by children’s caregivers and has been found to be responsive to treatment of ECC and to discriminate between children with various levels of caries experience [8,17]. ECOHIS, consists of 13 items, nine and four of which consider the impact of child’s oral health on child’s- and the family’s daily activities, respectively [8]. The child impact section (CIS) includes four domains; child symptom, function, psychology, self-image and social interaction. Two domains are included in the family impact section (FIS); parental distress and parental function. Details of the ECOHIS development and validation in its original English language version are reported elsewhere [8]. In light of the Tanzanian and Ugandan health policy prioritizing children below 5 years as target groups for oral health care services and considering the rising profile of childhood disability, ECOHIS is worthy consideration, by identifying groups vulnerable for impaired OHRQoL and by providing information that might aid in planning of health care policy initiatives [2].

### Purpose

The purpose of the present study was to assess the psychometric properties of an OHRQoL measure containing items emanating from the Child--and Family impact sections of the ECOHIS scale focusing 6–36 months old infants in Manyara region, Tanzania and Kampala district, Uganda. Specifically, the internal consistency, test -retest reliability and discriminative validity (i.e. the ability to differentiate the construct being measured from other similar constructs) of translated and culturally adapted versions of a Child- and a Family impact scores were evaluated. It was hypothesized that the Child- and Family impact scores would discriminate between children with and without clinically defined dental problems as well as between caretakers evaluating child’s general- and oral health condition as good and bad.

## Method

### Manyara site

A cross-sectional Reproductive and Child Health Services (RCHS)-based study was conducted in Manyara region, northern Tanzania from August 2010 to January 2011. Recruitment of children and their primary caretakers took place at Haydom Lutheran Hospital, HLH, and its 20 mobile outreach community service sites in Mbulu, Hanang and Babati districts of Manyara region. HLH is a 400-bed hospital owned by the Mbulu Diocese of the Evangelical Lutheran Church in Tanzania, incorporated fully into the National Health Plan [18]. Manyara region has a unique mix of ethnic groups as it is occupied by four sub Saharan African language groups; Hadzabe (Khoisan speaking people), Iraqwi (Cushitic), Datoga (Nilotic) and the group of Nyiramba, Nyaturu, Nyisanzu and Sukuma (Bantu speaking). The region is populated with a predominately poor rural population with low literacy rate [19]. The fluoride content in drinking water is about 3.0 mg F/L [20].

In collaboration with the government authorities through District Health Management Teams (DHMT) and other Voluntary Agencies, HLH has taken responsibility for an extensive outreach programme covering Reproductive and Child Health Services (RCHS). This RCHS includes one post with daily activities at the hospital in addition to 20 community posts, visited once a month by car on a rotating basis. The community outreach posts are not health facilities but may be any building available in the respective villages. According to the 2002 population and housing census in Tanzania, the RCHS outreach programme covered 6 out of 54 villages in Hanang, 3 out of 81 villages in Babati and 12 out of 70 villages in Mbulu, serving respectively, 4790, 1538 and 7910 children below 5 years of age [21]. During the project period, 21 RCHS outreach posts were visited 3–5 times on a rotating basis, recruiting approximately 10–14 caretaker-child pairs per visit. All caregiver-child pairs who were resident in the catchment areas of the RCHS posts and who satisfied the inclusion criteria were invited to participate in the study. The inclusion criteria were mother or primary caregiver of children aged 6–36 months attending for immunization and/or growth monitoring. Mothers were the primary target respondents, but in case of mothers’ absence, the primary caregiver was recruited. Out of 1250 caregiver-child pairs approached, 1221 agreed to participate (total response rate 97.7%, range 94-99%). A sample size (n = 1221) of this magnitude is sufficient to the pre-calculated sample of 810 caregiver-child pairs, assuming a prevalence of Child- and Family impact scores of 50%, a margin error of 5%, confidence level of 95% and a design effect of 2 [22]. Another 5% was added to the sample size to account for non responses and children to be excluded for being the second eligible child of the same mother or caregiver. A sample size of 1221 caregiver/child pairs was also satisfactory assuming a difference of child impact of 0.10 between groups with and without dental caries, a significance level of 5%, a power of 90% and a design factor of 2. Permission to carry out the study was granted by Medical Research Coordinating Committee of Ministry of Health and Social Welfare in Tanzania and the Ethical Research Committee in Norway (REK VEST). Informed written and/or verbal consent was obtained from all participating caretakers.

### Kampala site

A cross-sectional Mother and Child Health Clinic (MCH)-based study was conducted in Kampala district from June to October, 2007. Kampala covers an area of 197 km^2^ and has a population of 1.2 million of whom 18% are under 5 years. Kampala has an overall literacy rate of 75 % [19]. It is administratively divided into 5 divisions, two of which, Nakawa (42.5 km^2,^ total population in 2008 300,000) and Makindye (40.6 km^2^, total population in 2008 380,000), constituted the study areas. The districts have drinking water with fluoride content about 0.3 mgF/L [23]. One non-governmental (Kibuli) and one governmental (Naguru) MCH care facility were purposely selected in Makindye and Nakawa, respectively. Both facilities have large catchment areas and include community outreach clinics for the provision of child immunization. The inclusion criteria were caregivers with children aged 6–36 months attending the Kibuli and Naguru clinics for immunization and/or growth monitoring. All caregiver-child pairs who attended the clinics during the study period and who satisfied the inclusion criteria predefined for the study were eligible for participation. Out of 831 caregivers approached, 816 agreed to participate (response rate 82%). For a more detailed description of the calculation of sample size and sampling procedure and ethical issues, see [24].

### Interview

The interview schedule applied at both sites was constructed in English and translated into Luganda and Kiswahili, the main languages in Uganda and Tanzania. Kiswahili is the national official language in Tanzania spoken proficiently by almost 95% of the population. Single words of the Kiswahili interview was translated into Iraque, Datoga, Nyaturu and Nyisanzu languages, when deemed necessary during the interview. The interview schedule was translated in several steps; from English into local languages by bi-lingual Kiswahili/English and Luganda/English professionals, and then back translated to English by independent translators. Project staff at both sites reviewed the interview schedule for semantic, experiential and conceptual equivalence to the source version. Sensitivity to culture and selection of appropriate words were considered. Considering the young age of the children, and the local cultural contexts considered, 4 items of the original ECOHIS were deemed less appropriate and thus removed from the interview. The interview schedule was piloted among caregivers of preschool children to evaluate the quality of the translations in terms of comprehensibility, readability and relevance to assess face validity. Owing to poor comprehensibility of the FIS section in Manyara, this scale was deemed inappropriate to use and consequently removed from the Kiswahili interview.

At both sites, an OHRQoL measure based on selected items from the original ECOHIS instrument was administered in the field by trained locally recruited research assistants. The interviews were performed face to face with primary caretakers before their children underwent oral clinical examination.

Owing to the infrequent nature of oral problems, the caregivers were asked to consider the child’s whole life span. *Child impact score* was assessed using 5 of the original 9 questions in the CIS section of ECOHIS and covering the four sub-domains; child- symptoms, - function, -psychology, - self-image and child social interaction. Care givers were asked “Have your child (NAME) ever had toothache/symptoms from teeth and gums”, “Has your child ever been irritable and frustrated (cried)-, had trouble sleeping-, had difficulty eating-, avoided playing because of dental problems?” Responses were given as 0= no 1= yes, 2= I do not know. Dummy variables (0=no, 1=yes) were summarized (range 0–5) and dichotomized into 0= “no child impacts” and 1= “at least one child impact” after recoding all “Don’t know” categories to missing. *Family impact score* was assessed using the original 4 questions from the FIS section of the original ECOHIS instrument, covering two domains of family distress and family function. “How often have you or the other parent- because of child’s dental problems; taken time off from work, been upset, felt guilty and had financial problems”? Response categories were rated on a 5- point Likert scale; 0= never to 5= every or almost every day. Each item was dichotomized into 0= never/hardly never experienced family impact and 1= experienced family impact occasionally, often or very often. Dummy variables were summarized (range 0–4) and dichotomized into 0= no family impacts and 1= at least one family impact. *Perceived child health andoral health status* was measured by asking “Generally speaking – how would you describe the general health status/oral health status of Name (your child)?” Responses were given as (1) very good, (2) good, (3) bad and (4) very bad and subsequently dichotomized into good (category 1+2) and bad (category 3+4) for use in multiple variable analyses. *Teething symptom status* was assessed by asking “Did Name experience the following symptoms (gum irritation, fever, loss of appetite, diarrhea, increased salivation, vomiting, convulsions, coughing) during his/her tooth eruption? Responses was given as (1) “yes”, (0) “no”. A sum symptom score was created (range 0–8) and dichotomized yielding 0= less symptoms (score ≤3) and 1= more symptoms (score >3). *Socio-demographic variables* were assessed in terms of age, sex and caregivers education. Family wealth was assessed as an indicator of socio-economic status according to a standard approach in equity analysis [25]. Durable household assets indicative of family wealth (i.e. radio, television, telephone, refrigerator, lantern, cupboard, bicycle, motor cycle, car, boat) were recorded as (1) “available and in working condition” or (0) “not available and/or not in working condition.” These assets were analyzed using principal components analysis, PCA. The first component resulting from this analysis was used to categorize households into four approximate quartiles of wealth ranging from the 1^st^ quartile (least poor) to the 4^th^ (poorest).

### Clinical oral examination

Clinical examinations were carried out by one trained dentist at both study site (JK in Kampala and RM in Manyara), whereas trained assistants recorded the observations. Children were examined in knee to knee position using a dental mirror and natural light. Visible plaque was recorded initially as 1= present and 0=absent. Teeth were cleaned and dried by sterile gauze and inspected for ECC and LEH using disposable dental mirrors. Dental caries was assessed on fully and partially erupted teeth according to the World Health Organization criteria [26] and calculated in terms of decayed, filled and missed teeth. Linear enamel hypoplasia, LEH, was recorded on the buccal surfaces of each tooth present according to the criteria described by the developmental defects of Enamel (DDE) index proposed by FDI [27]. Experience with LEH was recoded as present (DDE>0) and absent (DDE=0). In Manyara, duplicate oral examinations including interviews of 81 child were caregiver pairs 3 weeks apart gave Cohen’s kappa for assessment of dental caries and LEH at tooth level in the range 0.85-1.0 and were 0.97 for the dmft. In Kampala, duplicate examinations of 24 child/caregiver pairs 2 weeks apart gave kappa statistics of 1.0, 0.8 and 1.0 regarding the prevalence of caries, presence of visible plaque and number of teeth erupted.

### Statistical analyses

Statistical Package for Social Sciences (SPSS) version 17.0 was used for data analysis (SPSS Inc., Chicago, IL, USA). Since study subjects were clustered within RCHS posts and MCH clinics, this cluster effect was considered in the data analysis to avoid overestimation of the precision of the estimates. In this study, the design effect of clustering was adjusted for using Complex sample in SPSS. Internal consistency reliability scales was examined using Cronbach’s alpha. Test-retest reliability analysis was performed using kappa statistics and Intra class correlation coefficients, ICC. Discriminative validity was examined by comparing the distribution of scores between groups with various levels on clinical indicators and teething symptoms and by comparing the distribution of the scores between groups with various levels of reported child oral health- and general health condition. Bivariate analyses were conducted using cross-tabulations and chi-square statistics. The overall Family- and Child impact scores were not normally distributed, but due to the large sample size, comparison between clinical groups and groups reporting good and bad health were performed using One-Way ANOVA test. To aid interpretation of mean differences in Child and Family impact scores, effect sizes (ES) were calculated as the mean difference between groups divided by the pooled standard deviation. The widely accepted thresholds of 0.2, 0.5 and 0.8 were used to define small, moderate and large effect sizes [28]. Multiple variable analyses were performed using standard logistic regression (SLR) for dichotomous outcomes with odds ratios, OR, and 95% Confidence intervals (CI) and Poisson regression for count data outcomes with rate ratios (RR) and 95% CI calculated. As some respondents had missing values on some variables the numbers presented in the tables might vary slightly.

## Results

### Sample characteristics

Table 1 depicts the categories and percentage distribution of socio-demographics-, clinical-, and self- reported health- and oral health related variables at each study site. As shown in Table 1, among the caregivers, 94.6% and 98.6% were mothers in Kampala and Manyara. Their mean ages were 24.7 (sd= 4.7) and 28.3 (sd= 6.5), respectively. Most children had at least one tooth erupted (above 80%). The ECC prevalence (dmft>0) amounted to 18.1% in low fluoride Kampala and 3.7% in high fluoride Manyara.

**Table 1:** **Frequency distribution of socio-demographics,clinical and non-clinical variablesand their categories inKampala (n=816) and Manyara(n=1221) study sites**

**Variables**	**Categories**	**Kampala % (n)**	**Manyara % (n)**
Relationship to child	Mother	94.6 (772)	98.6 (1204)
	Father/caregiver	5.3 (44)	1,4 (17)
Age of parent/caregiver	Less or equal to 24 yr	54.3 (443)	33.8 (403)
	≥25 yr	45.7 (373)	66.2 (789)
Sex of child	Boy	50.7 (414)	50.5 (616)
	Girl	49.3 (402)	49.5 (605)
Age of child	6-12 month	45.5 (371)	29.6 (362)
	13–24 months	29.7 (242)	50.9 (621)
	25–36 months	24.9 (203)	19.5 (238)
Father’s education	No formal	7 (49)	25.6 (309)
	Completed primary and higher	93 (654)	74.4 (899)
Marital status	Single/widow	14.6 (114)	8.1 (99)
	Married	85.4 (667)	91.9 (1122)
Teeth present	Absent	11.2 (91)	16.6 (203)
	Present	88.8 (725)	83.4 (1018)
Symptoms during teeth eruption	0-3 symptoms	54.5 (360)	47.7 (539)
	>3 symptoms	45.5 (301)	52.3 (590)
Caries experience (dmft>0)	Yes	18.1 (148)	3.7 (45)
	No	81.9 (668)	96.3 (1176)
Linear hypoplasia	Yes	13.7 (112)	7.9 (97)
	No	86.3 (704)	92.1 (1124)
Reported child oral health	Good	67.5 (551)	93.1 (1137)
	Bad	32.5 (265)	6.9 (84)
Reported child health	Good	77 (628)	22.7 (277)
	Bad	23 (188)	77.3 (944)

### Descriptive statistics and reliability

As shown in Table 2, child impacts related to difficulties eating (function dimension) and crying (psychological dimension) were most frequently reported by caretakers at both study sites with rates varying from 7.6% to 8.3% and from 32.5% to 36.5% in Manyara and Kampala, respectively. Family impacts related to taking time off from work (36.2%) and financial impacts to the family (38.2%) were the impacts most frequently reported in Kampala. At both sites, child impacts were most frequently reported for the older age groups. Taken time off from work and having financial problems because of the teeth and mouth problems were more frequently reported for children aged 25–36 months compared with their younger counterparts aged 6–12 months (Table 2). The mean Child impact scores were 0.3 (sd 0.9) and 1.1 (sd 1.5) in Manyara and Kampala respectively. The mean Family impact score in Kampala was 1.2 (sd 1.3). The total OHRQoL score (sum of Child and Family impact scores) averaged to 2.1 (2.7) (not in Table). Totals of 10.2%, 5.9% and 33.7% reported Family impacts only, Child impacts only and both Family- and Child impacts, respectively. In Manyara, kappa statistics for the Child impact items ranged from 0.65 to 0.89. In Kampala, kappa statistics for the Child-and Family impact scores ranged from 0.24-0.91 (not in Table). Table 3 shows the internal consistency reliability coefficients and the ICC and 95% confidence interval (CI) for the Child impact score across study sites and for the Family impact score and the total OHRQoL score in Kampala.

**Table 2:** **Frequency distribution of Childimpact and Family impactscores by age andsex in Manyara andKampala**

***Manyara***	**6-12 Month % (n)**	**13-24 Month % (n)**	**25-36 Month % (n)**	**Boys % (n)**	**Girls % (n)**	**Total % (n)**
**Child impact**						
How often has your child had pain in the teeth or mouth	3.6 (13)	6.1 (38)	8.0 (19)	6.3 (39)	5.1 (31)	5.8 (70)
How often has your child …… because of teeth or mouth problems?						
Been irritable and frustrated (cried)	5.5 (20)	7.4 (46)	11.3 (27)*	8.3 (51)	6.9 (42)	7.6 (93)
Had trouble sleeping	2.5 (9)	4.5 (28)	6.3 (15)	4.2 (26)	4.3 (26)	4.3 (52)
Had difficulty eating some foods	5.8 (20)	8.4 (52)	11.8 (28)*	8.3 (51)	8.3 (50)	8.3 (101)
Avoid playing	3.3 (12)	4.0 (25)	6.3 (15)	4.4 (27)	4.1 (25)	4.3 (52)
**Kampala Child impact**						
How often has your child had pain in the teeth or mouth	5.0 (18)	6.7 (15)	18.4(35)**	6.9 (27)	10.7 (41)	9.0 (68)
How often has your child …… because of teeth or mouth problems?						
Been irritable and frustrated(cried)	28.2 (101)	39.3 (88)	48.4 (92)**	33.3 (130)	39.5 (151)	36.5 (281)
Had trouble sleeping	11.2 (40)	18.8 (42)	21.1 (40)**	15.4 (60)	16.2 (62)	16.2 (122)
Had difficulty eating some foods	25.4 (91)	35.3 (79)	43.2 (82)**	31.5 (123)	33.8 (129)	32.5 (252)
Avoid playing	13.7 (49)	20.5 (46)	24.2 (46)**	16.9 (66)	10.7 (41)	18.6 (107)
**Family impact**						
How often have you or another family member						
…… because of your child’s teeth or mouth problems?						
Taken time off from work	28.5 (102)	40.2 (90)	44.7 (85)**	34.1 (133)	37.7 (144)	36.2 (277)
Been upset	19.6 (70)	26.8 (60)	25.8 (49)	21.3 (83)	25.1 (96)	22.8 (179)
Felt guilty	8.4 (30)	9.4 (21)	12.1 (23)	8.5 (33)	10.7 (41)	8.7 (71)
Financial problems	30.4 (109)	43.3 (97)	47.4 (90)**	36.2 (141)	40.6 (155)	38.2 (296)

* P<0.05, **P<0.01.

**Table 3:** **Internal consistency and testretest reliability analysis forthe Child impact andFamily impact scores inManyara and Kampala**

	**Cronbach’s alpha**	**ICC (95% CI)**
**Manyara**		
Child impact	0.83	0.96 (0.94 -0.97)
**Kampala**		
Child impact	0.84	0.47 (0.09-0.73)
Family impact	0.79	0.74 (0.48-0.88)
OHRQoL total	0.84	0.70 (0.22-0.41)

### Discriminative validity

In Manyara, 21.4% versus 12.8% (p<0.001) of caretakers who reported bad and good child oral health confirmed any Child impact (Child impact score e >0). Corresponding figures in Kampala were 57.4% versus 34.5% (p<0.001). Totals of 55.4% versus 39.7% (p<0.001) of caretakers’ reporting bad and good child oral health had Family impact score>0. Effect sizes with respect to mean differences between groups ranged from 0.1 to 0.3. Spearman’s correlation between Child- and Family impact scores in Kampala was 0.66 (p<0.001). Multivariable logistic- and Poisson regression analyses adjusting for potential confounding variables revealed statistically significant associations in the expected direction between the Child-and Family impact scores and caretakers’ reported Child health- and oral health. Child and Family impacts were most frequently experienced among those who reported bad child health and oral health (Table 4). In Manyara, multiple logistic regression revealed that the odds ratios of reporting Child impacts were 1.8 (95% CI 1.0-3.4) and 2.2 (1.3-3.4) among those who confirmed LEH and teething symptoms, respectively. In Kampala, the odds ratio for reporting Child impacts were 2.3 (95% CI 1.3-3.9), 1.7 (95% CI 1.1-2.5), 1.6 (95% CI 1.2-2.3) and 2.7 (95% CI 1.3-5.8) among those who confirmed presence of teeth, LEH, teething symptoms and tooth bud extractions, respectively (Table 5). In Kampala, the odds ratios for reporting Family impacts were 2.7 (95% CI 1.5-4.7), 1.5 (95% CI1.1-2.1) and 4.6 (95% CI 2.0-10.7) if reporting LEH, teething symptoms and toothbud experience, respectively. Corresponding results were achieved using Poisson regression analyses.

**Table 4:** **Discriminative validity Child impactand Family impact scoresby self- reported oralhealth and health status**

**Manyara Child impact**		**% (n)**	**Mean (sd) [ES]**^**a**^	**Adjusted OR (95% CI)**^**b**^	**Adjusted RR (95% CI)**^**c**^
Reported oral health	Bad	21.4 (18)	0.5 (1.3) [0.1]	1	2.0 (1.5-2.8)
	Good	12.8 (145)*	0.3 (0.9)*	0.5 (0.2-0.9)	
Reported health	Bad	14.7 (139)	0.3 (1.0) [0.1]	1	2.0 (1.5-2.8)
	Good	8.7 (24)**	0.2 (0.7)**	0.5 (0.3-0.8)	
**Kampala Child impact**					
Reported oral health	Bad	57.4 (101)	1.8 (1.8) [0.3]	1	1.8 (1.6-2.19
	Good	34.5 (213)**	0.9 (1.4)**	0.4 (0.3-0.6)	
Reported health	Bad	47.4 (120)	1.5 (1.7)[0.2]	1	1.4 (1.2-1.6)
	Good	35.9 (194)**	1.0 (1.5)**	0.7 (0.4-0.8)	
**KampalaFamily impact**					
Reported oral health	Bad	55.4 (143)	1.6 (1.4) [0.3]	1	1.7 (1.5-2.0)c
	Good	39.7 (217)**	0.8 (1.2)**	0.4 (0.2-0.5)	
Reported health	Bad	55.4 (143)	1.3 (1.4)[0.2]	1	1.3 (1.1-1.5)c
	Good	39.7 (217)**	0.9 (1.3)**	0.5 (0.4-0.7)	

^a^ Effect size.

^b^ adjusted for age of child, age of caregiver and child sex.

^c^ reference category: the last category by default.

**p<0.001, *p<0.05.

**Table 5:** **Discriminative validity: Child impactscores by clinical andself-reported oral health indicators**

**Manyara Child impact**		**% (n)**	**Mean (sd) [ES]**^**a**^	**Adjusted OR (95% CI)**^**b**^	**Adjusted RR (95% CI)**^**c**^
Caries experience	No	13.0 (154)	0.2 (0.9) [0.4]	1	0.6 (0.3-0.8)
	Yes	20.0 (9)	0.4 (1.2)	1.9 (0.9-4.5)	
Teeth present	No	7.4 (15)	0.1 (0.6)** [0.1]	1	0.5 (0.3-0.8)
	Yes	14.5 (148)*	0.3 (1.0)	2.0 (0.8-5.2)	
Hypoplasia	No	12.7 (143)	0.3 (0.9)*[1.0]	1	0.6 (0.4-0.8)
	Yes	20.6 (20)*	0.5 (1.1)	1.8 (1.0-3.4)	
Teething symptoms	No	9.0 (56)	0.2 (0.7) [0.1]|	1	0.5 (0.3-0.6)
	Yes	17.8 (107)**	0.4 (1.1)**	2.2 (1.3-3.4)	
**Kampala Child impact**					
Caries experience	No	37.3 (242)	1.0 (1.5) [0.2]	1	0.9 (0.7-1.1)
	Yes	49.7 (72)*	1.5 (1.7)*	1.2 (0.7-1.8)	
Teeth present	No	22.0 (20)	0.5 (1.2) [0.3]	1	0.5 (0.3-0.7)
	Yes	41.9 (294)**	1.2 (1.6)**	2.3 (1.3-3.9)	
Hypoplasia	No	37.4 (256)	1.1 (1.5) [0.1]	1	0.8 (0.6-0.9)
	Yes	53.7 (58)**	1.5 (1.7)*	1.7 (1.1-2.5)	
Teething symptoms	No	37.9 (132)	1.1 (1.5) [0.1]	1	0.7 (0.6-0.8)
	Yes	50.5 (147)**	1.5 (1.6)**	1.6 (1.2-2.3)	
Experience tooth bud extraction	No	38.3 (290)	1.1 (1.6) [0.3]	1	0.6 (0.4-0.8)
	Yes	68.6 (24)**	2.1 (1.8)**	2.7 (1.3-5.8)	
**Kampala Family impact**					
Caries experience	Sound	42.6 (281)	1.3 (1.3) [0.04]	1	0.9 (0.8-1.2)
	Decayed	54.5 (79)**	1.2 (1.4)	1.3 (0.9-2.0)	
Teeth present	No	23.3 (21)	0.5 (1.1) [0.2]	1	0.5 (0.3-0.6)
	Yes	47.5 (339)**	1.1 (1.4)**	2.7 (1.5-4.7)	
Hypoplasia	No	43.9 (304)	1.0 (1.3) [0.03]	1	1.0 (0.8-1.2)
	Yes	50.5 (56)	1.1 (1.3)	1.1 (0.7-1.6)	
Teething symptoms	No	43.7 (156)	1.0 (1.6) [0.1]	1	0.7 (0.6-0.9)
	Yes	55.7 (165)	1.4 (1.4)*	1.5 (1.1-2.1)	
Experience tooth bud extraction	No	43.0 (330)	1.0 (1.3) [0.4]	1	0.6 (0.4-0.7)
	Yes	81.1 (30)**	1.9 (1.3)**	4.6 (2.0-10.7)	

^a^ Effect size.

^b^ adjusted for child age, caregiver’s age and sex.

^c^ reference category: the last category by default.

* P<0.05, **P<0.01.

## Discussion

The present study is the first large population based survey focusing OHRQoL of preschool children below 5 years of age in Tanzania and Uganda. When comparing the Manyara study group with the 0-4- year- old child population in Mbulu, Babati and Hanang districts on some socio-demographic markers, it seems reasonable to suggest that the participants were representative of the under 5 year child population resident in those areas. In Kampala, the sampling method makes the external validity more questionable. However, the overall response rate was good and the number of missing items limited. This suggests that the Kampala study group for whom there are complete data is representative of caregiver/child pairs living in the catchment areas of the MCH clinics in Makindye and Nakawa districts.

When administered in face to face interviews, thus compensating for difficulties in interpretation that caregivers might suffer, the Luganda version of the Child impact and Family impact scores and the Kiswahili version of the Child impact score showed good psychometric properties, indicating satisfactory applicability to children 6–36 months old and their caretakers in the areas investigated. Despite the limitation of this study that the original CIS of ECOHIS was shortened by four items not considered appropriate in the socio-cultural contexts investigated, the internal consistency reliability was excellent, whilst considering the thresholds of Cronbach’s alpha value of 0.70 [29]. Moreover, the figures obtained compare favorably with Cronbach’s alpha values reported for the American, French, Chinese, Brazilian and Farsi sub-scale sections of ECOHIS [8,11,12,14,15]. In test-retest reliability, the ICC values suggested moderate to good consistency when compared to a threshold limit of 0.60 [29]. The ICC values observed in this study (0.47-0.96) are comparable to those obtained with the Chinese version of the original ECOHIS [12], but the ICC for the Child impact score in Kampala was below those reported in the English and French studies of ECOHIS [8,11]. The moderate value of the Child impact reproducibility score in Kampala (0.47) might be attributed to the young age of the study group, suggesting that rapid changes in health- and oral health status might have occurred between the test and retest sessions.

All participating caretakers completed the Child impact interview adding support to the face validity of its Luganda- and Kiswahili version. After having decided to remove four items from the original ECOHIS that were deemed less appropriate for the group of toddlers considered in this study, there were no indications from the academic reference groups or from the pilot study that the relevance of any of the remaining items was low in the two study sites. This suggests that caretakers of 6–36 months old children were capable of understanding the translated items without altering their meaning and that they are comparable with the corresponding items of the original English inventory. Notably, since only 5 of 9 items of the original CIS section were retained in the Child impact score investigated, the feasibility of using this east African subscale in cross-national comparisons is restricted. Whereas all participating caretakers completed the four items of the Family impact score in Kampala, adding support to the face validity of its Luganda version, caretakers in Manyara had difficulties interpreting the Kiswahili version of this score. Consequently, its relevance was considered low in the rural Tanzanian context. Notably, the OHRQoL instrument employed in this study deviates from the original ECOHIS in various aspects, the number of items used, the wording of items and the type of measurement scale. This, the Child- and Family impact scores assessed should be considered a starting point of a subsequent process of identifying items to be considered when studying OHRQoL of children and their families in the East African cultural context.

Discriminative validity was confirmed across study sites in that both the Child- and Family impact scores varied systematically and in the expected direction between children reported to have good and bad general- and oral health conditions. Discriminative validity testing on known clinical groups was expected to reveal higher impact scores among children with- than without oral diseases. As shown in Table 5, when clinical indicators were adjusted for potentially confounding factors, caries experience approached statistical significance in Manyara but did not remain a significant covariate of the Child impact score in Kampala. In contrast, LEH emerged as a strong positive clinical covariate of Child impact scores across both study sites. The lack of a strong significant relationship with ECC might be attributed to the low disease prevalence of this study group, particularly in Manyara. In fact the responsiveness to change of the original ECOHIS has been shown to be rather limited when the inventory was applied in samples with low levels of oral problems. An alternative explanation might be poor discriminative validity of the Child impact score due to the process of translating items into African languages. In Kampala, Family impacts of dental caries seemed to be more serious in the younger than in the older age groups. Previous studies have shown that the original ECOHIS as well as its sub sections are able to discriminate between children with and without severe caries experience, but not between children with and without other oral diseases [13,30] . Using the Child OIDP among Tanzanian and Ugandan school aged children revealed more impaired OHRQoL among children with than without dental caries experience [30]. To some extent this comparison between two OHRQoL measures developed for use in children highlights the relative discriminative ability of the Child and Family impact scores in the Tanzanian and Ugandan cultural contexts.

Caregiver’s perception of the oral impacts on children’s- and the family’s activities, with reference to the child whole life span, was substantial in Kampala, amounting to 39.6% and 44.8%, respectively. Among US children aged 5 years, the corresponding figures where 58.3% and 45.6% [8]. A substantial difference was observed between the two study sites regarding the prevalence of child impact with 39.6% and 13% of caregivers reporting impacts in Kampala and Manyara, respectively. The latter figure is lower than those observed in most other studies using the original ECOHIS inventory [8,11,15]. This low prevalence might be due, in part, to the very young study group considered and to the fact that whereas the present study focused community based samples, most previous studies have utilized individuals recruited in dental health care hospitals, suffering particular oral health problems. The higher prevalence of Child impacts seen in Kampala as compared to Manyara is in line with Kampala children having a less healthy profile generally. Moreover, the Kampala children were older and had a higher number of erupted teeth. Diversity in cultural contexts and demographic characteristics as well as the fact that different birth cohorts were investigated, might have contributed to the difference in the prevalence of oral disease and OHRQoL between the study groups. The present finding suggesting that Kampala parents reported more Family impacts (44%) than Child impacts (39%) is opposite what was reported among Brazilian preschool children [13]. Considering that this study focused on caregiver child pairs attending reproductive health care facilities for immunization and weight monitoring in response to an invitation and not for treatment purposes, the strength of this study is the possibility of extrapolating the findings beyond the health care office setting to the general population.

## Conclusion

To our knowledge there are yet few studies that have considered the impact on quality of life from oral diseases among preschool children in the general population of sub-Saharan Africa. The present findings suggest that the translated and culturally adapted versions of the Child and Family impact scores demonstrated acceptable internal consistency reliability and reproducibility whereas the discriminative validity was more ambiguous. This suggests that the Child and Family impacts scores should be developed and tested further among Kiswahili and Luganda speaking caretakers of 6–36 months old infants who are inhomogeneous in terms of culture, prevalence of oral problems as well as demographic characteristics. This study suggests that toddlers could benefit from screening their oral health status from early ages on. Policy makers should consider integrating oral health care interventions into routine reproductive health care plans.

## Competing interests

The authors declare that they have no competing interests.

## Authors’ contribution

RM: principal investigator, designed the study, collected the data (Manyara site), performed the statistical analyses, and wrote the manuscript. AB: participated in the design of the study, provided valuable guidance in the data collection and was actively involved in statistical analyses and manuscript writing. KM: provided valuable guidance in statistical analyses. AN: designed the study, guided the statistical analyses and the manuscript writing. All authors have red read and approvexd the final manuscript.
